# Comprehensive FISH Probe Design Tool Applied to Imaging Human Immunoglobulin Class Switch Recombination

**DOI:** 10.1371/journal.pone.0051675

**Published:** 2012-12-14

**Authors:** Jakub Nedbal, Philip S. Hobson, David J. Fear, Rainer Heintzmann, Hannah J Gould

**Affiliations:** 1 Randall Division of Cell and Molecular Biophysics, King’s College London, London, United Kingdom; 2 MRC and Asthma UK Centre in Allergic Mechanisms of Asthma, Guy’s Hospital, London, United Kingdom; 3 Division of Asthma, Allergy and Lung Biology, Guy’s Hospital, London, United Kingdom; 4 Institute of Photonics Technology, Jena, Germany; 5 Institute of Physical Chemistry, Friedrich Schiller University, Jena, Germany; University of North Carolina at Charlotte, United States of America

## Abstract

We present a web engine boosted fluorescence *in-situ* hybridization (webFISH) algorithm using a genome-wide sequence similarity search to design target-specific single-copy and repetitive DNA FISH probes. The webFISH algorithm featuring a user-friendly interface (http://www.webfish2.org/) maximizes the coverage of the examined sequences with FISH probes by considering locally repetitive sequences absent from the remainder of the genome. The highly repetitive human immunoglobulin heavy chain sequence was analyzed using webFISH to design three sets of FISH probes. These allowed direct simultaneous detection of class switch recombination in both immunoglobulin-heavy chain alleles in single cells from a population of cultured primary B cells. It directly demonstrated asynchrony of the class switch recombination in the two alleles in structurally preserved nuclei while permitting parallel readout of protein expression by immunofluorescence staining. This novel technique offers the possibility of gaining unprecedented insight into the molecular mechanisms involved in class switch recombination.

## Introduction

Fluorescence *in-situ* hybridization (FISH) is a cytogenetic tool for detection and localization of specific nucleic acid sequences in chromosomal, cellular or tissue preparations [1], [2]. It is used in medical diagnosis, species identification or cellular function studies [3]. Fluorescent probes, complementary to the desired target sequences, are annealed under slightly denaturing conditions to nucleic acids in fixed and permeabilized cells or chromosome spreads. For single-copy DNA targets, the probe typically consists of a mixture of short (100–300 bases) overlapping single-strand DNA fragments derived from the target sequence ranging from a few kilobases up to the entire length of a chromosome. If parts of the probe are similar to multiple sites in the genome, it becomes cross-specific, causing ambiguity in the identification and localization of its target. This phenomenon however facilitates FISH on repetitive targets such as chromosomal satellites, which can be detected with a conventional DNA probe or a single oligonucleotide species [4].

**Figure 1:**
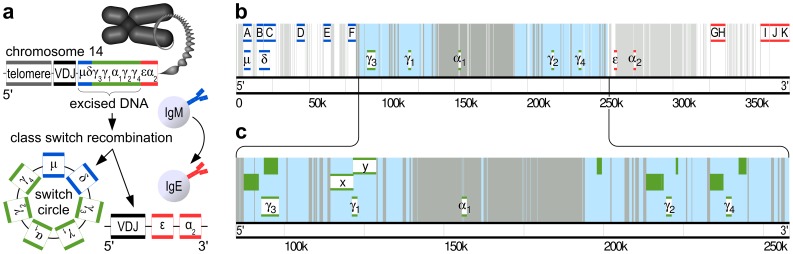
Human immunoglobulin heavy chain constant region genes and specific FISH probes. (**a**) The relative positions of the nine immunoglobulin heavy chain constant genes, 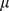 to 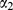, in tandem array on chromosome 14 are illustrated by blue, green and red horizontal double bars. They are located near the telomere of human chromosome 14, downstream of the heavy chain variable, diversity and joining (VDJ) genes. Switching from the production of IgM to IgE, is caused by class switch recombination. It involves the juxtaposition of the replacement heavy chain constant region gene to the VDJ genes through the deletion of the intervening chromosomal DNA that forms a switch circle. (**b**–**c**) The genes (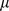 to 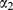) are superimposed on the background of colors representing the chromosomal DNA similarity identified by webFISH. The *white* background highlights unique sequences, which lack similarity to the rest of the genome. The *gray* background denotes sequences similar more than one genome sequences. The *cyan* background marks a locally repetitive subset of the *gray* similar sequences. These sequences share similarity exclusively within the chromosomal DNA segment in (**c**), but not with any other part of the genome. (**b**)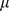-specific (blue horizontal double bars, A–F) and -specific (red horizontal double bars, G–K) single-copy FISH probes were identified within the contiguous stretches of unique (*white* background) sequences exceeding 4 kilobases in length. (**c**)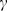-specific repetitive FISH probes (green horizontal double bars, x&y) were designed to bind only locally repeating sequences (including the adjacent green filled bars) but not any other part of the genome. The combined length of the -specific FISH probes is 14.3 kb but overall it binds 37.0 kb of sequence.

The design of single-copy FISH probes has already been improved and simplified by software tools predicting the binding based on the lack of similarity between the target sequence and the rest of the genome. These tools select optimal single-copy targets for FISH [5] or pick a tiling array of specific oligonucleotide probes [6], [7]. The advantages of these approaches include probe specificity for a single target [5]–[7], probe reproducibility [6], [7] and consistent labeling efficiency [7]. Oligonucleotide-based FISH strategies [6], [7] (Stellaris, Biosearch Technologies, Novato, CA, USA; SureFISH, Agilent, Santa Clara, CA, USA) are especially promising due to their reproducibility and economy. While possible in principle, these methods currently do not support the design of FISH probes for locally repetitive targets, such as those in the immunoglobulin heavy chain locus (Figure 1b). This led us to the development of an online tool webFISH for straightforward and flexible identification of all potential specific single-copy and/or repetitive FISH probes inside a user-specified chromosomal sequence (http://www.webfish2.org/, Supplementary Software).

In human, the immunoglobulin heavy chain locus comprises nine heavy chain constant region genes (Figure 1a) downstream of an ensemble of variable, diversity and joining (VDJ) genes. The constant region genes give rise to immunoglobulins of nine different classes having specific functions and anatomical distributions. The expression of a particular immunoglobulin class is governed by class switch recombination. In this process, one of the heavy chain constant region genes is juxtaposed to the C-terminal VDJ genes by deletional recombination, forming an excision switch circle (Figure 1a). Class switch recombination is an irreversible process that can occur repeatedly until the immunoglobulin heavy chain repertoire is exhausted in a given cell. The regulation and execution of class switch recombination is dependent on the combined activity of protein complexes, chromosomal restructuring and DNA transcription [8].

Aberrant class switch recombination can result in the development of allergy, immunodeficiencies, infectious diseases or B cell neoplasia. Of particular interest is the switching to immunoglobulin E (IgE), which is normally tightly regulated and occurs much less frequently than switching to other classes. IgE regulation is perturbed with dire consequences in immunodeficiencies such as the Hyper IgE syndrome, Omenn syndrome, Wiscott-Aldrich syndrome and DiGeorge syndrome [9]. Stimulation of heavy-chain switching events also promotes aberrant switching to cause B cell neoplasia [10] and immune response to infectious diseases, such as parasite [11] and rhinovirus [12] infections. However, the most common link is to allergic disease, which has become an epidemic in the developed world [13]–[15]. To better understand the pathogenesis of these conditions, a technique is needed to distinguish class switch recombination to IgE or IgG on individual chromosomes of a primary B cell population while permitting a parallel readout on local recruitment, activation or repression of proteins and transcription of DNA.

Examination of the class switch recombination is typically done by polymerase chain reaction (PCR) or sequence analysis of the newly formed switch junctions in the chromosome or the resulting switch circles and their transcripts [16] (Figure 1a). However, these methods are usually only applicable to cell populations. When employed in single cells they can neither discriminate between the class switch recombination in the two immunoglobulin alleles nor can they be combined with other techniques to allow readout of associated molecular processes. Delayed readout of class switch recombination is commonly achieved by flow cytometry or ELISA investigation of the subsequently expressed immunoglobulin class. This approach is subject to a delay consistent with the time required for the new immunoglobulin class expression and the lasting presence of the previously expressed immunoglobulin class.

Class switch recombination on both immunoglobulin alleles of individual cells was initially detected by sequencing the restriction digest fragments of the genomic DNA from immortalized B cells [17]. In primary B cells this was first performed using fiber FISH [18], [19] to detect the associated DNA deletion from the immunoglobulin locus. Cells were lysed on glass slides and DNA unwound and released from histones by stepwise treatment with concentrated salt solution, propidium iodide and ultra violet (UV) irradiation [20]. The DNA fibers were used as targets of multiple FISH probes labeled with spectrally distinctive fluorophores. The total length of the examined immunoglobulin locus DNA corresponded to the length of the pattern of fluorescence signals resembling “beads on a string”. This pattern uniquely identified the locus amongst the rest of the genome. The difference in the fluorophore distribution pattern of germline and recombined DNA revealed the length and the identity of the deleted region, giving information on the class of last recombination. The utilized FISH probes that cross-hybridize with the non-target DNA due to sequence similarities present in the immunoglobulin locus [21] did not interfere with this measurement. However, they would be unsuitable for studies on interphase nuclei aiming to preserve the 3D architecture of the chromosomes and the native DNA-protein interactions. BAC-derived and commercial probes were hybridized in interphase nuclei to detect DNA deletion in class switch recombination based on the decrease, but not a complete loss of the fluorescence signal [22]. This approach could not differentiate between IgE and IgG switch and could not reliably distinguish class switch recombination from low hybridization efficiency.

Our goal was to perform interphase nuclei FISH for reliable detection of the DNA deletion associated with class switch recombination to one of the IgGs or IgE. In our approach, a complete loss of binding of one probe would indicate recombination to IgG and of two probes would signify recombination to IgE. When combined with RNA FISH or immunofluorescence, this technique would enable simultaneous examination of nuclear localization and recombination state assessment for both immunoglobulin alleles of individual primary B cells in combination with recruitment of proteins, their post-translational modification or DNA transcription.

To obtain specific FISH probes for interphase nuclei FISH studies of class switch recombination, we have developed and validated the webFISH algorithm by designing and experimentally testing single-copy and repetitive FISH probes for the IgM–IgD, IgG
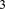
–IgG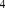 and IgA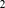 chromosomal regions. Similarly challenging sites for FISH probe design that could benefit from webFISH capabilities include, for example, the immune receptor (Figure S1), the olfactory receptor or the ribosomal RNA genes.

## Results

### WebFISH Principle

WebFISH is available as an online resource (http://www.webfish2.org/) and in the form of a source code (https://github.com/webfish/). It aids the design of FISH probes for DNA of species with known genome sequence. The analysis is configurable to suit individual experimental needs. WebFISH delivers a pair of primers for the production of each identified FISH probe and provides a graphical output of the analysis (Figure 1b–c) alongside a report containing the details about the optimized PCR primers and the amplicon sequences. In webFISH, the user-supplied query sequence is aligned using Megablast [23] to the unmasked genome sequences of the target organism obtained from the most recent release of Ensembl [24]. Identified Megablast alignments are subject to an adjustable minimum bit score requirement (200 by default), which poses a threshold for the required sequence similarity. It avoids short similarities between the query sequence and the genome, which are unlikely to result in efficient FISH probe hybridization. Sequences similar to more than one site in the genome (Figure 1b–c, gray or cyan background) are distinguished from the remaining unique sequences (Figure 1b–c, white background). Contiguous stretches of the unique sequences provide targets for specific single-copy FISH probes. Nearly contiguous stretches of similar sequences are identified (Figure 1c) and analyzed for the presence of exclusively locally occurring repeats (cyan background). These regions provide targets for locus-specific repetitive FISH probes. PCR primers are automatically designed using Primer3 [25]. These enable FISH probe production either by direct synthesis in a PCR with fluorescently labeled nucleotides or by cloning of the amplicons into plasmids, which then serve as substrates for labeling reactions. The resulting FISH probes form a tiling array covering contiguous stretches of both single-copy and repetitive sequences. The targets for the repetitive FISH probes are chosen to achieve the highest possible cumulative length of their binding sites (Figure 1c, green filled bars). The program is designed to run with a default set of parameters specified in a commented configuration file delivered with its results. This file can be altered to modify the bit score threshold in the similarity search, the permissible gaps in the nearly contiguous similar sequence regions, the limits for the PCR amplicon lengths, the parameters for the primer pair search and the graphical output parameters.

### Immunoglobulin Heavy Chain Gene Specific FISH Probes

WebFISH is aimed at designing FISH probes for complicated targets. A proof of principle experiment was performed on the immunoglobulin heavy chain locus containing nine constant region genes surrounded by regulatory and recombinatory regions. These genes undergo deletional class switch recombination in B cells (Figure 1a) in order to change the N-terminal constant region domains of the expressed immunoglobulin, thereby modifying its immunological function and anatomical distribution. The mutual similarity of the genes and the associated sequences precludes manual design of specific FISH probes [21] (demonstrated in [18]). However, webFISH designed three sets of specific FISH probes targeting different parts of the investigated sequence. Two single-copy FISH probe sets shown in Figure 1b are regions A–F in blue at the 5′ end and G–K in red at the 3′ end. The center of the sequence binds a set of repetitive x&y FISH probes depicted in green in Figure 1c. WebFISH has been also used for the design of FISH probes specific for the mouse immunoglobulin constant region genes, the human immunoglobulin 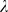 light chain genes and the human T cell receptor 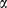, 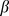, 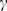, and 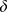 genes (Figure S3). Since these probes have not been experimentally tested, they merely demonstrate the wide applicability of webFISH.

### Imaging Immunoglobulin Class Switch Recombination

**Figure 2:**
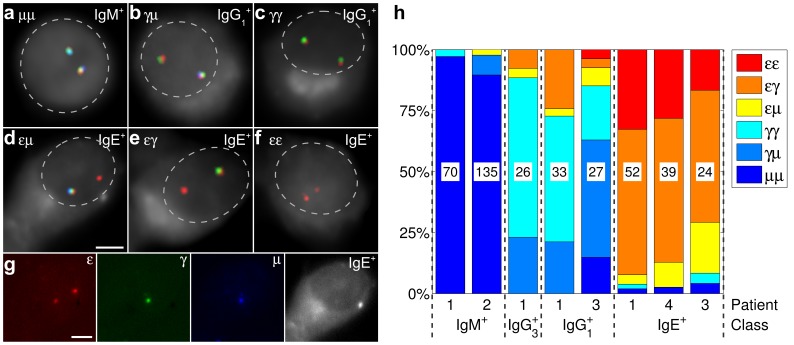
Class switch recombination states detected by FISH. (**a**–**g**) IgM, IgG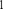 or IgE-expressing cells represented by combinations of “μ” (A–F, blue), “
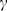
” (x&y, green) or “

The three FISH probes (each with its specific color) were hybridized simultaneously in naïve human primary B cells cultured in conditions promoting class switch recombination from expression of the IgM class to one of the IgG classes or IgE [26]. The outcome of the class switch recombination depends on the extent of the chromosomal DNA deletion. This prevents hybridization of one or two of the FISH probes when switch to IgG or IgE has occurred, respectively. Simultaneous binding of all three probes, thus indicates the lack of any class switch recombination and the expression of the IgM class. The loss of binding of the 5′ probe A–F (Figure 1b, blue) alone suggested recombination to one of the IgG classes. The lack of both the 5′ A–F (blue) and the central x&y (green) probes was the hallmark of recombination to the IgE class (Figure 1f). In the same cells, the expressed class of the immunoglobulin was assessed by simultaneous IgG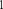-, IgG
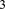
- or IgE-specific immunocytochemical staining. The sort of IgM-expressing cells achieved 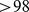
% purity and therefore all cell in the specimen were considered IgM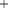 (Figure S2). The class switch recombination states of both immunoglobulin alleles were assessed from the type of co-localizing FISH probes, as explained above, in the groups of cells expressing the same immunoglobulin class (Table S2). The results from four different donors are summarized in Figure 2h. The majority (90–97%) of the pure IgM-expressing cells, which have not undergone any deletional recombination, bound all three FISH probes simultaneously in both alleles (Figure 2a). Similarly, most (92–97%) IgE-expressing cells did not bind both the 5′ probe A–F (blue) and the central x&y (green) in one allele. On the other allele it either remained non-recombined (Figure 2d,g), recombined to one of the IgG classes (Figure 2e) or the IgE class (Figure 2f). The IgG
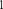
- or IgG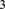-expressing B cells usually (70–88%) lacked the binding site to the 5′ A–F (blue) probe in one (Figure 2b) or both (Figure 2c) alleles as a result of the deletional class switch recombination occurring on single or both alleles, respectively. Yet, 11–27% of these cells lack binding of both 5′ A–F (blue) and central x&y (green) probes, resembling the IgE-expressing cells. This is likely due to ongoing expression of IgG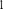 or IgG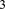 in cells that have already switched to IgE. We observed considerable variation in the relative frequency of switching in different B cell donors, but the results are consistent with the orderly switching from 5′ to 3′ in the tandem array of immunoglobulin heavy-chain gene sequences on human chromosome 14. The results demonstrate the specificity of the webFISH-designed probes. They bring direct evidence for the occurrence of asynchronous class switch recombination on the immunoglobulin alleles.

” (G–K, red) FISH probes. Gray background stains (**a**) DNA in a population of pure IgM-expressing cells or (**b**–**g**) the expressed immunoglobulin class in cells enriched for IgG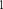 or IgE expression. Dashed lines approximate the nuclear outline. Details about the applied image processing algorithm are presented in “Materials and Methods”. (**g**) Decomposition of panel (**d**) into raw images of individual spectral channels (raw 2D data for **a**–**f** are presented in Figure S1 and raw 3D data are available in Supplementary Software, https://github.com/webfish/). Scale bar, 2 µm. (**h**) Each of the four classes, IgM, IgG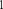, IgG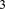 and IgE, was studied in one to four patients (1–4). From patient 4 only IgE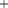 cells were examined. The number of scrutinized cells from each patient and the patient identifiers are inside and below the graph bars, respectively.

## Discussion

The presented webFISH algorithm designs all permissible specific single-copy and repetitive FISH probes for the user-supplied query sequence. It features an intuitive online interface combined with maximum flexibility through the optional use of a configuration file. With the ever increasing number of sequenced genomes and the quality of their assembly, the scope of webFISH application will keep increasing in the future. However, webFISH probe search reliability will remain dependent on the accuracy and completeness of the target species genome assembly.

WebFISH overcomes the aforementioned limitations of previous methods of FISH arising from the presence of repetitive sequences in the genome. These normally interfere with unambiguous detection and localization of specific nucleic acid sequences in chromosomal, cellular or tissue preparations described in the Introduction. FISH probes designed using webFISH offer unprecedented insight into the molecular mechanisms of class switch recombination by overcoming their restrictions. More generally, WebFISH can be used to test potential FISH probes derived from stock plasmids (e.g. bacterial artificial chromosomes, cosmids, fosmids) for their specificity to a single genomic site and thereby guide the selection of the most suitable clones. WebFISH functionality could be extended to RNA FISH probe search for transcription studies by performing single-strand alignment against the cDNA library of the examined species.

The presented technique still remains limited by the requirement for the use of naïve IgM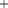 B cells cultured in conditions promoting switching to IgE or IgG. (Conditions required for switching to IgA were not used in the present work.) In a total B cell population, IgA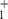 or IgA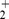 recombination cannot be distinguished from IgG or IgE recombination. Furthermore, false-positive detection of a recombination event can result from poor FISH staining and therefore weak or absent fluorescent signal. Most likely, this would happen for IgG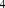 and less so for IgG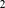 recombination mistaken for IgE recombination. Due to the deletional recombination, the binding region for the 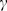-specific probe in chromosomes recombined to IgG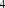 or IgG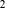 is only 1.8–6.6 and 7.2–12.6 kilobases, respectively.

Despite these limitations, the technique enables direct readout of class switch recombination from individual chromosomes, characterizing a cell population at the level of single cells. It can be combined with immunofluorescence staining to allow parallel detection and intra-cellular localization of the studied proteins and their interactions with DNA. It should equally well enable parallel RNA transcription detection by RNA FISH performed with the same cells [27]. Alternatively, the method could be employed to detect recombination to IgA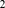 in cells cultured with TGF-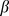.

Based on the presence of cells with both the same and different recombination states on their immunoglobulin alleles, we can infer that class switch recombination occurs on both alleles, albeit asynchronously. It remains unclear if the expressed allele switches always earlier than the excluded one. To examine this possibility, RNA FISH could be employed to distinguish the expressed allele in a subset of cells featuring single allele RNA-DNA FISH co-localization [28]. The technique can be combined with immunofluorescence staining for proteins associated with gene activation and silencing to understand the molecular mechanism of allelic exclusion and class switch recombination. When employed with cells cultured for variable periods, the timing of responsible protein association and recombination could be unraveled.

## Materials and Methods

### Ethics Statement

The study was approved by the the Guy’s Research Ethics Committee under the reference code 08/H0804/94. The Guy’s Research Ethics Committee is constituted in accordance with the Governance Agreements for Research Ethics Committees (July 2001) and complies fully with the Standard Operating Procedures for Research Ethics Committees in the UK. All child patients and their parents or legal guardians were informed about the purpose, effects and the voluntary nature of their participation in the study. The parents or legal guardians of all involved patients gave oral and signed consent to their participation. All subject data were handled anonymously.

### Probe Design

Human chromosome 14q32∶105947801–106327800 sequence, obtained from Ensembl containing all human immunoglobulin heavy chain constant region genes, was processed by webFISH versions 1.0 and 2.0 (Supplementary Software, https://github.com/webfish/). The utilized configuration files and sequences are included in the Supplementary Software. The algorithm automatically identified the lack of the ClaI restriction site 5′-ATCGAT-3′ in all of the proposed FISH probe targets. To allow later clonal screening by restriction digest, the ClaI restriction site was linked to the 5′ end of each of the 13 pairs of the designed primers (Table S1).

### FISH Probe Template Cloning

PCR was performed with BAC templates RP11-731F5 and RP11-417P24 (BACPAC Resources Center, Oakland, Ca, USA) using Long PCR Enzyme Mix (Fermentas, St. Leon-Rot, Germany) following the manufacturers guidelines. To increase their specificity, 4% (v/v) supplementary DMSO was added to reactions with primers J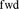:J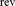, K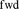:K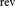 and x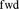:x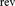, while reactions with primers H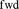:H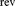 and I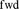:I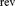 were performed with 1 M betaine (Sigma-Aldrich, Gillingham, UK). Products were purified by 0.6% agarose (Bioline Reagents, London, UK) gel electrophoresis and GeneJET™ Gel Extraction Kit (Fermentas) and cloned into bacterial expression vectors using a StrataClone PCR Cloning Kit (Agilent, Stockport, UK) following the manufacturer’s instructions. Cloning was verified by agarose electrophoresis restriction digest mapping and by forward and reverse sequencing with T3 and T7 primers (Eurofins MWG Operon, Ebersberg, Germany). Verified clones were transformed into XL1-Blue competent cells (Agilent) for storage and plasmid production.

### FISH Probe Synthesis

Plasmids were isolated from 250 ml overnight cultures by Plasmid Plus Maxi Kit (Qiagen, Crawley, UK). 50 µg of each vector was freed of salts by ethanol precipitation and linearized with KpnI (clone H) or NotI (all other clones). To obtain the pure DNA required for subsequent nick translation, a combination of phenol extraction, chloroform extraction, ethanol precipitation with three washes and silica matrix based purification with the QIAquick PCR Purification Kit (Qiagen) were utilized. Plasmids for production of a particular FISH probe (Table S1) were combined in equimolar amounts (55–70 pM). Nick translation was performed on 2 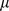
g of this substrate as previously described [29] using DNA Polymerase I (NEB, Hitchin, UK), dioxynucleotide triphosphate (dNTP) mix (GE Healthcare, Little Chalfont, UK), aminoallyl-dioxyuridine triphosphate (dUTP) (Invitrogen), DNAse I (Roche Applied Science, Burgess Hill, UK) and 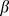-mercaptoethanol (Sigma-Aldrich). The product was denatured for 3 minutes at 95°C followed by rapid cooling on ice and resolved in 1.5% agarose gel electrophoresis into a smear of fragments ranging between 100 and 300 bases in length. It was purified using QIAquick PCR Purification Kit and concentrated by ethanol precipitation. ARES™ Kits (Invitrogen) were employed according to the recommended protocol to label the -specific, 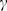-specific and 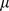-specific FISH probes with Alexa Fluor® 488, 594 and 647, respectively. The obtained FISH probes were purified using the QIAquick PCR Purification Kit into nuclease free water. The FISH probe “hybridization buffer” consisted of 3.3 ng
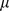
l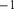 of each FISH probe, 500 ng
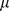
l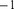 of salmon sperm blocking DNA (Rockland, Gilbertsville, PA, USA), 50 ng
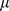
l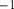 Cot-1 blocking DNA (Roche Applied Sciences), 5% (w/v) dextran sulphate (Sigma-Aldrich), 1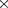 SSC and 50% (v/v) deionized formamide (Applied Biosystems, Warrington, UK).

### Cell Isolation and Culture

Human B cells were isolated from tonsils collected from patients undergoing routine tonsillectomies at the Evelina Childrens Hospital (Guy’s and St. Thomas’ NHS Foundation Trust). The patients’ parents or legal guardians gave informed written consent for participation in this study. Human tonsillar B cells were isolated as reported previously [30] by dissection, density gradient purification and T cell depletion with 2-(2-aminoethyl) isothiourea dihydrobromide (AET)-treated sheep erythrocytes. Naïve IgM
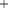
IgD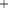 B cells were purified by positive selection for their IgD-expression using magnetic-activated cell sorting (MACS) [31]. Instructions supplied with the anti-phycoerythrin (PE) microbeads (Miltenyi Biotec, Bisley, UK) were followed with modifictions described below. Up to 5
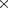
10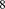 B cells were labeled for 45 minutes in the dark with 500 ng
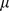
l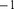 PE conjugated anti-human IgD antibody (Southern Biotech, Birmingham, AL, USA) in MACS buffer. They were washed, resuspended in 400 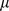
l of MACS buffer with 100 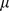
l of anti-PE microbeads for every 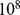 cells. Following another 45 minute incubation in the dark, they were purified using an LS column (Miltenyi Biotec) in VarioMACS™ magnetic stand (Miltenyi Biotec) according to manufacturer’s instructions. Cells were cultured with 240 I.U.

ml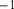 IL-4 (R&D Systems, Abingdon, UK) and 1 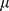
g

ml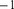 anti-human CD40 antibody G28-5 (produced in-house) as described previously [30]. IL-4 and CD40 cross-linking by the antibody provide the signals for class switch recombination to IgE and IgGs [26].

### Enrichment of IgG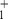, IgG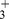 and IgE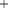 B Cells

Prior to enrichment, the B cells were cultured for 5 days in case of IgG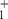 or IgG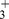 (IgG switch occurs earlier) and for 9 days for IgE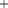 cells (IgE switch occurs later). 4
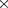
10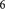 to 8
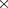
10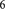 of these cells were pooled and depleted of dead cells by density gradient centrifugation [30]. Live cells were washed in MACS buffer for 20 minutes and labeled with either 1∶10 dilution of IgG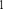 (Miltenyi Biotec), 1∶2000 dilution of IgG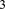 (Sigma-Aldrich) or 800 ng
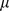
l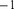 of IgE (Vector Labs, Peterborough, UK) class specific biotinylated anti-human antibodies. Cells were washed twice, and incubated for 20 minutes with 600 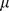
l of MACS buffer and 20 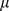
l of anti-biotin microbeads (Miltenyi Biotec). They were washed twice, and enriched using MS column (Miltenyi Biotec), according to the manufacturer’s instructions. All steps were performed on ice. The enrichment yielded purity of about 30% based on microscopic counting of cells stained with fluorescent class specific anti-human immunoglobulin antibodies (data not shown).

### Fluorescence *in situ* Hybridization

2
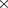
10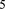 freshly isolated IgM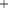, IgG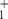, IgG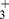 or IgE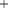 cells in 50 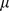
l culture medium were incubated for 5 minutes at 37°C on acid washed and poly-L-lysine (Sigma-Aldrich) coated coverslips (Science Services, Munich, Germany). They were swelled for 45 seconds in 0.3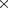 PBS (Invitrogen) and immediately fixed and permeabilized as previously described [32]. Prior to hybridization, IgG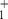, IgG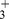 or IgE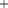 cells were labeled with biotinylated class-specific anti-human immunoglobulin antibodies as mentioned above. Denaturation and hybridization of the FISH probes in “hybridization buffer” (see “FISH Probe Synthesis”) were performed in a Hybaid OmniSlide Thermal Cycler (HBOSBB, Thermo Scientific). A two minute denaturation at 76°C was followed by an overnight (12–16 hour) incubation at 37°C. Ramping between the starting ambient, denaturation and hybridization temperature steps was carried out at 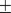
0.5°C

s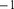. Post hybridization washing was performed as described previously [29] in a circulating water bath. Prior to mounting, nuclei of IgM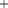 cells was stained for 10 minutes with 100 ng

ml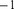 Hoechst 33258 DNA stain (Invitrogen). IgG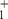, IgG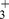 or IgE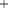 cells were instead incubated for 1 hour in wash buffer containing 5% (v/v) normal goat serum (Invitrogen), followed by 15 minute labeling with 200 ng

ml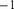 Alexa Fluor 405-conjugated streptavidin (Invitrogen) solution in wash buffer containing 1% normal goat serum. Finally, cells were were washed three times and mounted in Prolong Gold® (P36934, Invitrogen). They were stored at 4°C for a one night up to a week before imaging to allow hardening of the mounting medium.

### Image Acquisition and Processing

Samples were inspected with a Zeiss Axio Observer wide field fluorescence microscope utilizing a 63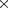/1.4 NA Plan-APO objective and 1.6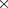 magnifying tube lens (Optovar). Fluorophore excitation was achieved with 405, 488, 561, and 635 nm lasers and laser-line specific filters. An iXon3 885 EM-CCD camera (Andor, Belfast, UK) with a conventional readout was used for the image acquisition. Purification of the IgM-expressing cells achieved high purity of the cell population (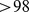
% by flow cytometry, Figure S2c), therefore all cells in the specimens were treated as IgM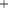. Only IgG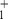, IgG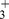 or IgE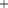 cells featuring bright patchy staining near the cell surface (Figure S2) were chosen for further analysis. The Alexa Fluor® 405 immunoglobulin staining was spectrally unmixed from the FISH probe images in the 488 nm and 594 nm excitation channels by a custom-written Matlab script (Supplementary Software, https://github.com/webfish/). FISH probe images of selected cells were merged into a three-color overlay. In these, the two brightest spots containing the -specific FISH probe focus were identified. The types of FISH probe foci co-localized in each of these spots were reported (Table S2). Typical images in Figure 2a–g were created by averaging one or two sections containing the two immunoglobulin allele images. To improve their clarity in print, the images were low-pass Gaussian filtered, thresholded for the brightest 2% image area and merged into four-color overlay (Supplementary Software, https://github.com/webfish/). The raw unprocessed images are in Figure S1.

### Supplementary Software

https://github.com/webfish/ The repository contains source code for webFISH version 1.0, webFISH version 2.0 and the image processing algorithm including the raw microscopy data for Figure 2 and Figure S2.

## Supporting Information

Figure S1
**Class switch recombination states detected by FISH, raw data.** IgM, IgG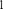 or IgE-expressing cells represented by combinations of “
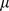
” (A–F, blue), “
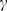
” (x&y, green) or “

” (G–K, red) FISH probes. Gray background staining shows **(a)** DNA or **(b**–**f)** the expressed immunoglobulin class. Dark patches inside FISH probes images are result of spectral unmixing of the immunoglobulin staining. Scale bar, 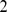
m.(PDF)

Figure S2
**Discrimination of cells expressing a specific immunoglobulin class.** **(a)** IgE-expressing cells are distinguished by bright spots of anti-IgE immunocytochemical staining near the cell surface inside the cytoplasm. **(b)** Cells not considered to be IgE-expressing are those lacking any distinctive signal or exhibiting a bright signal originating in the center of the cell. A similar discrimination was used to identify IgG
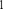
- and IgG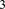-expressing cells with the help of appropriate immunoglobulin class-specific antibodies. Dashed lines approximate the nuclear outline based on the nuclear autofluorescence. Scale bar, 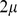
m. **(c)** The nave IgM
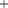
IgD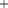 B cells were isolated to 99% purity verified by flow cytometry. The histograms of anti-human IgD labeling efficiency in populations of freshly isolated tonsillar B cells are in red and of the purified nave B cells used in the experiments are in blue. Due to the high purity, the cells did not require immunofluorescent staining. Instead all were regarded as IgM and IgD expressing.(PDF)

Figure S3
**Other possible applications for webFISH.** **(a)** The mouse immunoglobulin heavy chain constant region genes (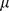, 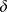, 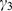, 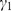, 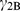, 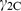,  and 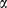, red) are formed by a smaller proportion of similar sequences compared to the human. This enables the design of specific single-copy (A–K, pink) FISH probes for the IgM, IgD, IgE, and IgA genes and repetitive (a–c, blue) FISH probes for the IgG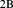 and IgG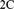 genes. **(b)** Human immunoglobulin 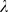-light chain single-copy (A–K, pink) and repetitive (a–c, blue) FISH probes were identified and displayed along with twelve of the 33 functional variable genes (shown in red V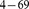, V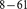, V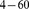, V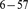, V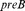, V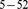, V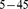, V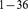, V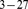, V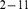, V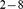, V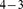) and two of the five constant region genes (shown in red C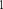, C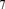) for reference. **(c)** TCR α and TCR 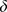 genes are largely composed of unique sequences offering numerous single-copy FISH probe targets (A–AE, pink). The TCR α locus is composed of a single functional constant region (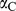, red), 67 joining (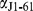, red) and 45 variable (shown in red 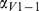, 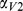, 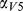, 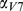, 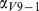, 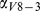, 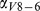, 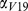, 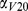, 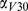, 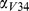, 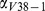 and 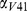) genes. The TCR 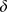 locus consists of one constant (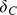), four joining, three diversity (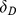) and eight variable (example 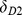) genes. Five of the variable genes are shared between α and 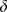 (shown in red 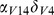 and 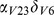). **(d)** TCR β genes are largely similar. Two contiguous similar regions with locally repetitive sequences (cyan background) were identified, each offering binding sites for specific repetitive (a–c, blue) FISH probes. Downstream unique sequences around the constant region genes (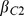) allow for single-copy FISH probe design (A–D, pink) The two orange regions emphasize chromosomal regions that have not been assembled into the genome sequence yet and therefore cannot be used for FISH probe search. There are 48 functional variable (shown in red 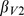, 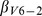, 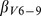, 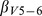, 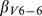, 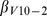, 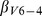, 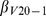, 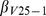, 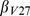 and 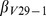) and two constant (shown in red 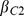) genes, each linked to one joining and six diversity genes (not shown). **(e)** TCR 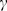 locus consists of two thirds of predominantly unique sequences harboring single-copy FISH probes (A–H, pink). The rest being locally repetitive sequences allowing design of repetitive FISH probes (a–c, blue). The TCR 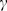 locus includes six functional variable (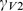, 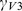, 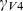, 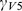, 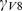 and 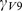, red), one constant (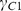, red) gene with its three joining genes (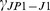, red) and a second constant (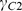, red) gene with its two joining genes (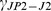, red).(PDF)

Table S1
**Primers.** PCR primers were designed to generate PCR products for cloning into vectors used in FISH probe production. Six pairs of primers are for the “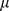-specific”, five pairs for the “-specific” probe and two pairs for the “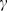-specific” FISH probe vectors. The primers are extended by a ClaI restriction endonuclease recognition site “atcgat” on their 5′ ends to enable screening for successful cloning. Primers A–H, x and y were designed with webFISH version 1.0 and primers I–K with webFISH 2.0. See Supplementary Software, https://github.com/webfish/. The expected product lengths are in the third column.(PDF)

Table S2
**Class switch recombination states and immunoglobulin expression.** Cells from four different donors (1–4) expressing immunoglobulin class IgD, IgG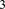, IgG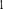 or IgE were assigned one out of six class switch recombination states (, 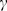, 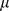, 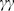, 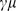 or 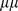). Remaining cells, which could not be unambiguously assigned a class switch recombination state due to insufficient or nonspecific FISH probe staining, were labeled “O”.(PDF)
