# Supplementary material for: Comprehensive FISH Probe Design Tool Applied to Imaging Human Immunoglobulin Class Switch Recombination
Source: PLoS One. 2012 Dec 14;7(12):e51675. doi: 10.1371/journal.pone.0051675 (PMC3522715; doi:10.1371/journal.pone.0051675)
Supplement: Figure S1 — Class switch recombination states detected by FISH, raw data. IgM, IgG or IgE-expressing cells represented by combinations of “” (A–F, blue), “” (x&y, green) or “” (G–K, red) FISH probes. Gray background staining shows (a) DNA or (b–f) the expressed immunoglobulin class. Dark patches inside FISH probes images are result of spectral unmixing of the immunoglobulin staining. Scale bar, m. (PDF) [file pone.0051675.s001.pdf]

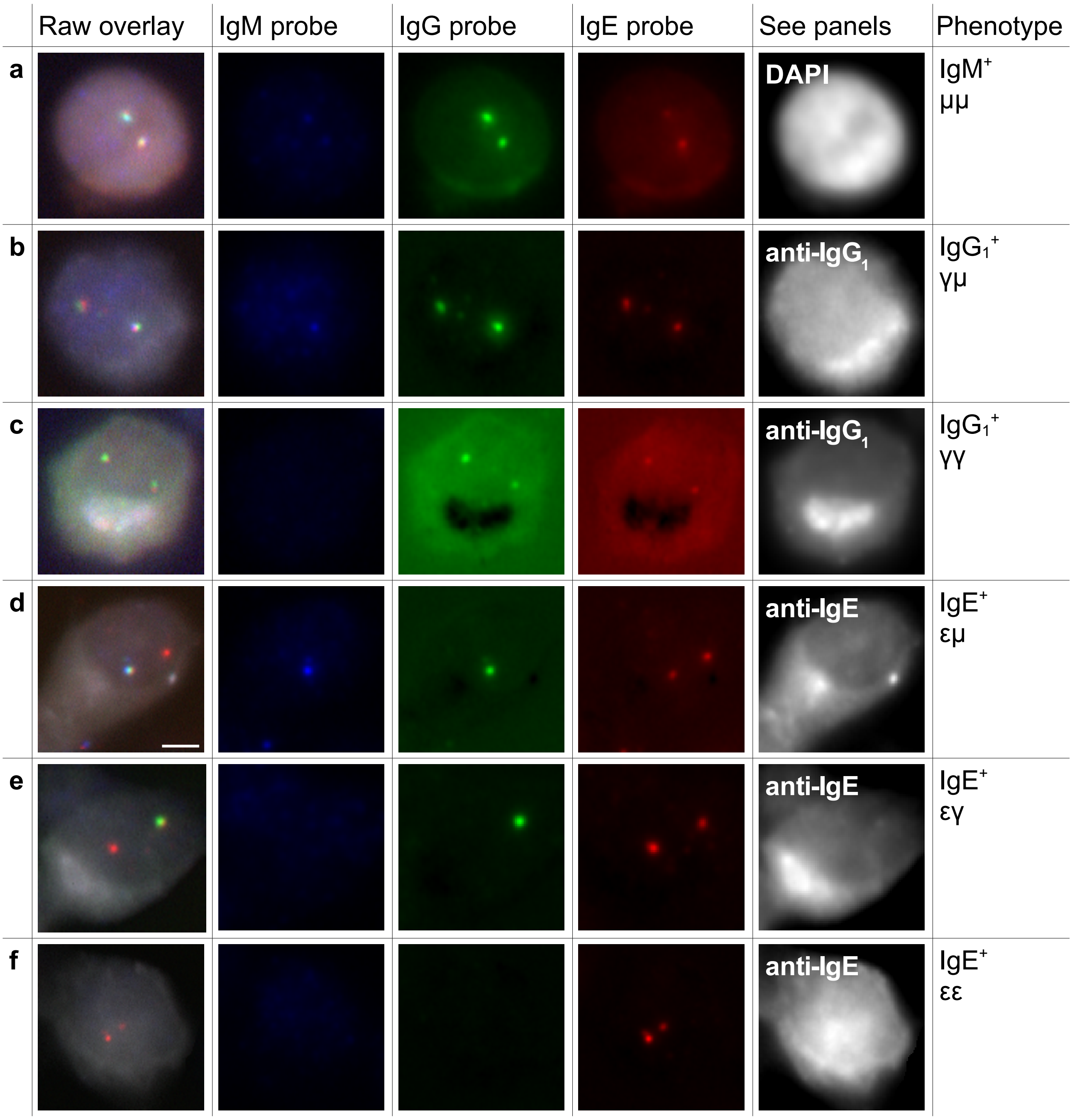

Raw overlay
IgM probe
IgG probe
IgE probe
See panels
Phenotype
a
DAPI
IgM^+
μμ
b
anti-IgG_1
IgG_1^+
γμ
c
anti-IgG_1
IgG_1^+
γγ
d
anti-IgE
IgE^+
εμ
e
anti-IgE
IgE^+
εγ
f
anti-IgE
IgE^+
εε
