# Supplementary figures and images for: Comprehensive FISH Probe Design Tool Applied to Imaging Human Immunoglobulin Class Switch Recombination
Source: PLoS One. 2012 Dec 14;7(12):e51675. doi: 10.1371/journal.pone.0051675 (PMC3522715; doi:10.1371/journal.pone.0051675)

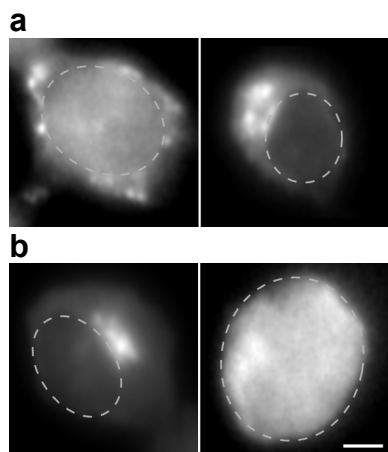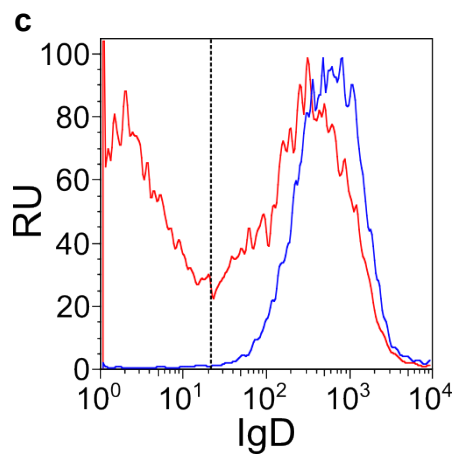

Supplement: Figure S2 — Discrimination of cells expressing a specific immunoglobulin class. (a) IgE-expressing cells are distinguished by bright spots of anti-IgE immunocytochemical staining near the cell surface inside the cytoplasm. (b) Cells not considered to be IgE-expressing are those lacking any distinctive signal or exhibiting a bright signal originating in the center of the cell. A similar discrimination was used to identify IgG- and IgG-expressing cells with the help of appropriate immunoglobulin class-specific antibodies. Dashed lines approximate the nuclear outline based on the nuclear autofluorescence. Scale bar, m. (c) The nave IgMIgD B cells were isolated to 99% purity verified by flow cytometry. The histograms of anti-human IgD labeling efficiency in populations of freshly isolated tonsillar B cells are in red and of the purified nave B cells used in the experiments are in blue. Due to the high purity, the cells did not require immunofluorescent staining. Instead all were regarded as IgM and IgD expressing. (PDF) [file pone.0051675.s002.pdf]

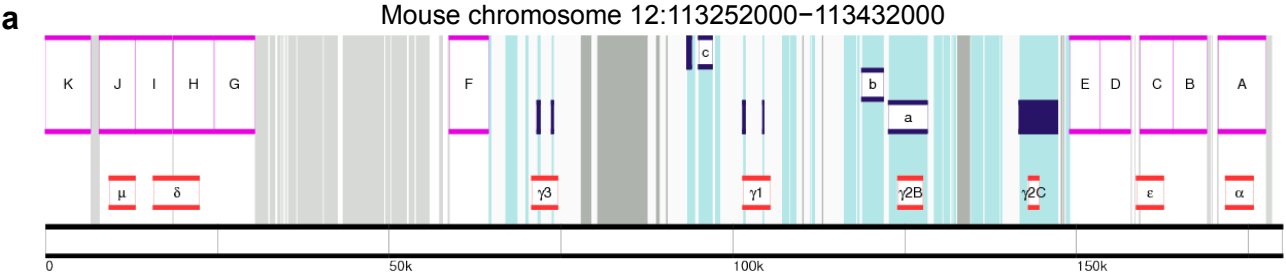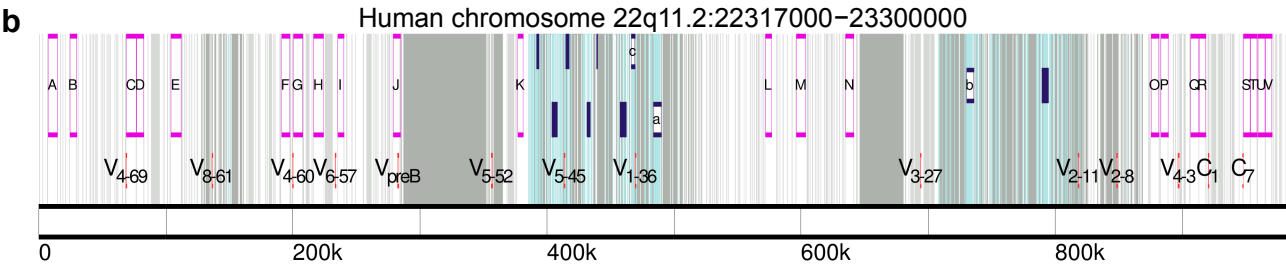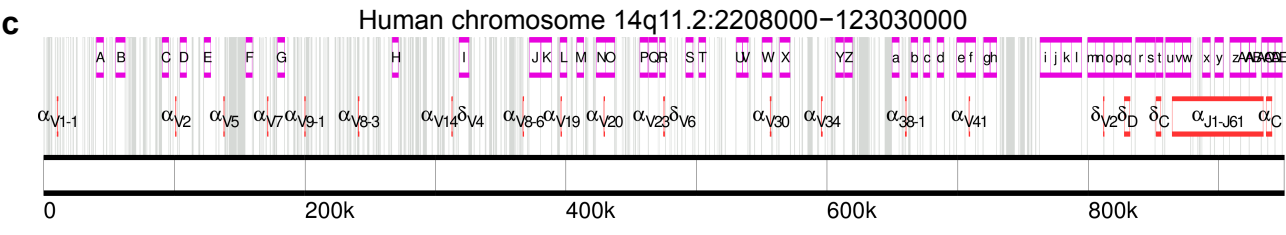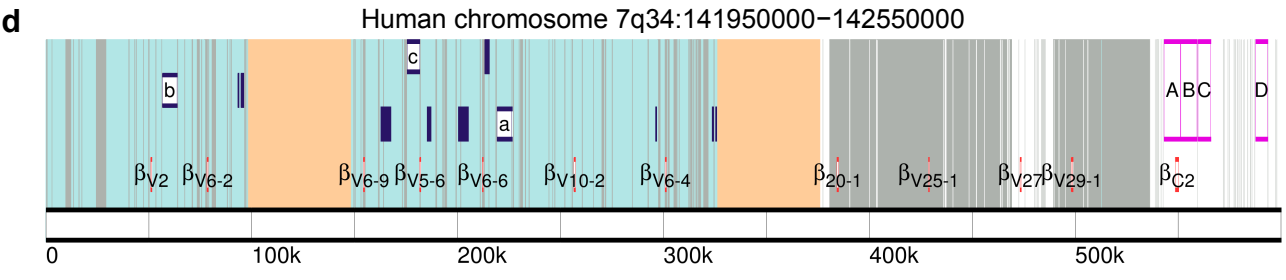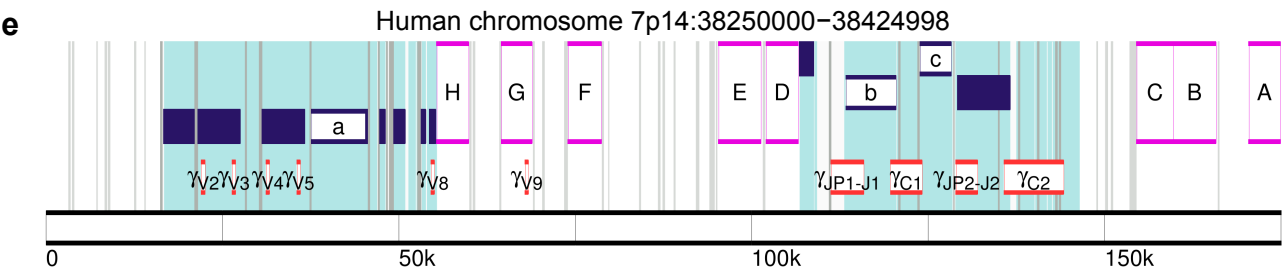

Supplement: Figure S3 — Other possible applications for webFISH. (a) The mouse immunoglobulin heavy chain constant region genes (, , , , , , and , red) are formed by a smaller proportion of similar sequences compared to the human. This enables the design of specific single-copy (A–K, pink) FISH probes for the IgM, IgD, IgE, and IgA genes and repetitive (a–c, blue) FISH probes for the IgG and IgG genes. (b) Human immunoglobulin -light chain single-copy (A–K, pink) and repetitive (a–c, blue) FISH probes were identified and displayed along with twelve of the 33 functional variable genes (shown in red V, V, V, V, V, V, V, V, V, V, V, V) and two of the five constant region genes (shown in red C, C) for reference. (c) TCR α and TCR genes are largely composed of unique sequences offering numerous single-copy FISH probe targets (A–AE, pink). The TCR α locus is composed of a single functional constant region (, red), 67 joining (, red) and 45 variable (shown in red , , , , , , , , , , , and ) genes. The TCR locus consists of one constant (), four joining, three diversity () and eight variable (example ) genes. Five of the variable genes are shared between α and (shown in red and ). (d) TCR β genes are largely similar. Two contiguous similar regions with locally repetitive sequences (cyan background) were identified, each offering binding sites for specific repetitive (a–c, blue) FISH probes. Downstream unique sequences around the constant region genes () allow for single-copy FISH probe design (A–D, pink) The two orange regions emphasize chromosomal regions that have not been assembled into the genome sequence yet and therefore cannot be used for FISH probe search. There are 48 functional variable (shown in red , , , , , , , , , and ) and two constant (shown in red ) genes, each linked to one joining and six diversity genes (not shown). (e) TCR locus consists of two thirds of predominantly unique sequences harboring single-copy FISH probes (A–H, pink). The rest being locally repetitive sequences al [file pone.0051675.s003.pdf]
