# Supplementary material for: Comprehensive FISH Probe Design Tool Applied to Imaging Human Immunoglobulin Class Switch Recombination
Source: PLoS One. 2012 Dec 14;7(12):e51675. doi: 10.1371/journal.pone.0051675 (PMC3522715; doi:10.1371/journal.pone.0051675)
Supplement: Table S1 — Primers. PCR primers were designed to generate PCR products for cloning into vectors used in FISH probe production. Six pairs of primers are for the “-specific”, five pairs for the “-specific” probe and two pairs for the “-specific” FISH probe vectors. The primers are extended by a ClaI restriction endonuclease recognition site “atcgat” on their 5′ ends to enable screening for successful cloning. Primers A–H, x and y were designed with webFISH version 1.0 and primers I–K with webFISH 2.0. See Supplementary Software, https://github.com/webfish/. The expected product lengths are in the third column. (PDF) [file pone.0051675.s004.pdf]

μ-Specific Probe Primers (5'→3')		Product Length
A_{fwd}	atcgatGGCTGCGCGAGGATGCAGTGAGG	5749
A_{rev}	atcgatTGCGTTCCCCATCACACCCACCC	
B_{fwd}	atcgatCCTCTCCCCATTGTGTTTTCCTGGTGCC	4165
B_{rev}	atcgatTGTGGGCAGGGTTGGCTCCC	
C_{fwd}	atcgatGGCACTGCATGATGACTACTGTTGC	8178
C_{rev}	atcgatACCAACCTAAGCACCCCTCAGC	
D_{fwd}	atcgatTCTCTTCCTTCTCACTTCTCCTTCTGGG	5467
D_{rev}	atcgatGGACCAAGCTCCCTCCACACC	
E_{fwd}	atcgatAGGCCACACTGCAAATCACACCTTGGC	4793
E_{rev}	atcgatACGCCCCAGGACTGTTGGCTGC	
F_{fwd}	atcgatACACCGGGGCCTATTCCTCCTACC	5059
F_{rev}	atcgatGGGCTGCTGTTGCCACCTTGGG	
**ε-Specific Probe Primers (5'→3')**		
G_{fwd}	atcgatCCCAGCGCGTGGGTCTGTTTGGG	5300
G_{rev}	atcgatGGGTGGTTACGACAGCAAGCTGGGC	
H_{fwd}	atcgatAGGGGACCACATCTCCTGGGGC	5283
H_{rev}	atcgatATGGGCTTCAGGGCGGGTGC	
I_{fwd}	atcgatTGCATATGGCCGGGCAGGAGGG	5873
I_{rev}	atcgatAGCGCACTTGGTCTCACGCGCC	
J_{fwd}	atcgatTCGGCCCTGCCTGAAGTGCG	6610
J_{rev}	atcgatAGCCAGTGATGACGGGCGGG	
K_{fwd}	atcgatAAAACCCCCGGGGCCTGACG	6748
K_{rev}	atcgatTCGCACTTCAGGCAGGGCCG	
**γ-Specific Probe Primers (5'→3')**		
x_{fwd}	atcgatTGGACGTGTCAGCACCCGGC	7556
x_{rev}	atcgatGCCCATTCTGGACGCTTCCCGTTCC	
y_{fwd}	atcgatACCTAGTGCCCAGGGGGATGC	7493
y_{rev}	atcgatACCTCTGGGGGCACAGCAGC	
