# Supplementary material for: Comprehensive FISH Probe Design Tool Applied to Imaging Human Immunoglobulin Class Switch Recombination
Source: PLoS One. 2012 Dec 14;7(12):e51675. doi: 10.1371/journal.pone.0051675 (PMC3522715; doi:10.1371/journal.pone.0051675)
Supplement: Table S2 — Class switch recombination states and immunoglobulin expression. Cells from four different donors (1–4) expressing immunoglobulin class IgD, IgG, IgG or IgE were assigned one out of six class switch recombination states (, , , , or ). Remaining cells, which could not be unambiguously assigned a class switch recombination state due to insufficient or nonspecific FISH probe staining, were labeled “O”. (PDF) [file pone.0051675.s005.pdf]

**Class**	**IgD⁺**		**IgG$_3$⁺**	**IgG$_1$⁺**		**IgE⁺**		
**Donor**	**1**	**2**	**1**	**1**	**3**	**1**	**4**	**3**
εε	0	0	0	0	1	12	11	4
εγ	0	0	2	8	1	31	23	13
εμ	0	3	1	1	2	2	4	5
γγ	2	0	17	17	6	1	0	1
γμ	0	11	6	7	13	0	0	0
μμ	68	121	0	0	4	1	1	1
O	0	0	0	2	0	2	1	2
